# Predicting long-term progression of Alzheimer’s disease using a multimodal deep learning model incorporating interaction effects

**DOI:** 10.1186/s12967-024-05025-w

**Published:** 2024-03-11

**Authors:** Yifan Wang, Ruitian Gao, Ting Wei, Luke Johnston, Xin Yuan, Yue Zhang, Zhangsheng Yu, for the Alzheimer’s Disease Neuroimaging Initiative

**Affiliations:** 1 Department of Bioinformatics and Biostatistics, School of Life Sciences and Biotechnology, Shanghai Jiao Tong University, 800 Dongchuan Road, Minhang District, Shanghai, 200240 China; 2 SJTU-Yale Joint Center for Biostatistics and Data Science, Shanghai Jiao Tong University, Shanghai, China; 3 School of Mathematical Sciences, Shanghai Jiao Tong University, Shanghai, China; 4 Clinical Research Institute, Shanghai Jiao Tong University School of Medicine, Shanghai, China

**Keywords:** Artificial intelligence, Deep learning, Alzheimer’s disease, Early diagnosis, Multimodal biomarkers

## Abstract

**Background:**

Identifying individuals with mild cognitive impairment (MCI) at risk of progressing to Alzheimer’s disease (AD) provides a unique opportunity for early interventions. Therefore, accurate and long-term prediction of the conversion from MCI to AD is desired but, to date, remains challenging. Here, we developed an interpretable deep learning model featuring a novel design that incorporates interaction effects and multimodality to improve the prediction accuracy and horizon for MCI-to-AD progression.

**Methods:**

This multi-center, multi-cohort retrospective study collected structural magnetic resonance imaging (sMRI), clinical assessments, and genetic polymorphism data of 252 patients with MCI at baseline from the Alzheimer’s Disease Neuroimaging Initiative (ADNI) database. Our deep learning model was cross-validated on the ADNI-1 and ADNI-2/GO cohorts and further generalized in the ongoing ADNI-3 cohort. We evaluated the model performance using the area under the receiver operating characteristic curve (AUC), accuracy, sensitivity, specificity, and F1 score.

**Results:**

On the cross-validation set, our model achieved superior results for predicting MCI conversion within 4 years (AUC, 0.962; accuracy, 92.92%; sensitivity, 88.89%; specificity, 95.33%) compared to all existing studies. In the independent test, our model exhibited consistent performance with an AUC of 0.939 and an accuracy of 92.86%. Integrating interaction effects and multimodal data into the model significantly increased prediction accuracy by 4.76% (*P* = 0.01) and 4.29% (*P* = 0.03), respectively. Furthermore, our model demonstrated robustness to inter-center and inter-scanner variability, while generating interpretable predictions by quantifying the contribution of multimodal biomarkers.

**Conclusions:**

The proposed deep learning model presents a novel perspective by combining interaction effects and multimodality, leading to more accurate and longer-term predictions of AD progression, which promises to improve pre-dementia patient care.

**Supplementary Information:**

The online version contains supplementary material available at 10.1186/s12967-024-05025-w.

## Introduction

Alzheimer’s disease (AD) is the leading cause of dementia, and the rising clinical demand for AD diagnosis and treatment places a growing strain on healthcare systems, particularly in the context of an aging population [1]. In recent years, early identification and intervention for AD have gained considerable attention. Patients in the pre-dementia stage, such as those with mild cognitive impairment (MCI), are expected to derive more benefit from potential treatments [2]. However, it is important to note that the causes and outcomes of MCI vary widely, and not all individuals with MCI will inevitably develop AD [3]. Therefore, accurate differentiation of MCI patients who will progress to AD is essential for targeted and preventive interventions.

Various biomarkers have been used for MCI conversion prediction. Structural magnetic resonance imaging (sMRI) is non-invasive and sensitive to brain atrophy [4–6]. Clinical assessment and neuropsychological testing are crucial components of current diagnostic criteria for probable AD [7, 8]. In addition, genome-wide association studies (GWAS) have identified a series of genetic variants associated with AD [9]. An effective combination of multimodal biomarkers complements each other and facilitates the early diagnosis of AD. However, the complex search for this optimal combination makes manual diagnosis by qualified experts time-consuming and expensive.

Recent years have witnessed a growing number of studies on automated MCI conversion prediction tools. Some concentrated on the short-term prediction that, despite promising results, had limited clinical relevance because it related to later interventions that fail to reverse the already existing neuronal loss [10–14]. In long-term prediction studies, some traditional machine-learning-based methods involved complex feature engineering, resulting in the omission of important pathological features [15–18]. End-to-end deep neural network (DNN) methods can offer solutions to these limitations and hold great promise for clinical decision support. But even with the full utilization of DNNs, models using only unimodal biomarkers are insufficient for MCI conversion prediction [19–21]. Several studies have developed multimodal DNN approaches to provide comprehensive insight into the disease progression [22–25]. While such studies have achieved convincing results, it is worth noting that, in theory, DNNs exhibit suboptimal performance when dealing with inputs that contain complex interactions, compared to data that can be structured as a composition of a series of layers, such as images [26, 27]. This points to the necessity for architectural enhancements in conventional multimodal DNNs to accommodate intra-modal and inter-modal interactions effectively.

Our study aimed to develop and validate a deep learning-based model with dual interaction modules to accurately predict the long-term conversion from MCI to AD using sMRI, clinical characteristics, and genetic polymorphism data. We also assessed model robustness across different clinical centers and imaging scanners as well as elucidated the contribution of multimodal biomarkers.

## Methods

### Participants

**Fig. 1:**
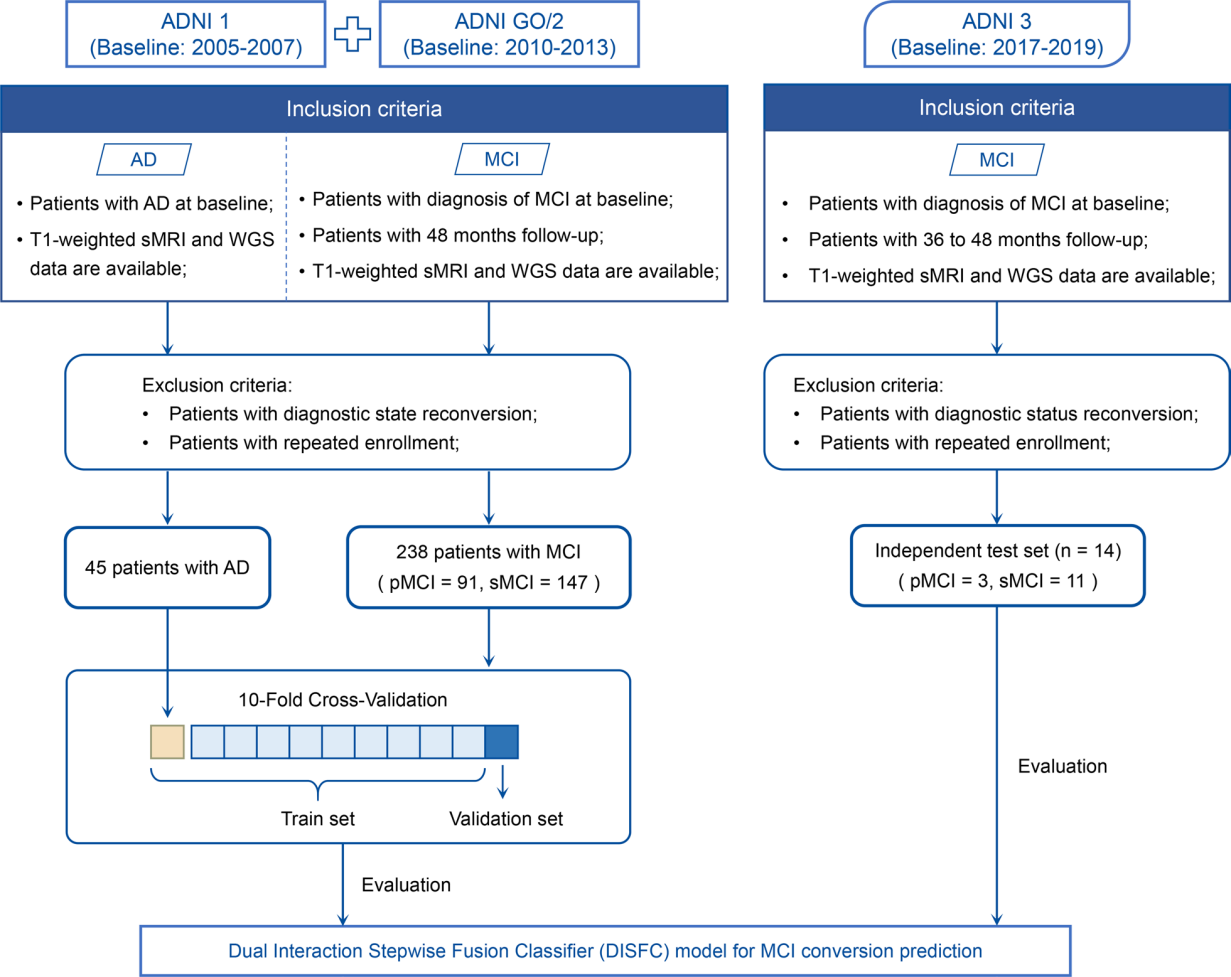
Flowchart of study design. The study comprised 238 subjects with MCI from ADNI-1 and ADNI-2/GO cohorts for cross-validation, and 14 subjects with MCI from ADNI-3 for an independent test. In addition, 45 subjects diagnosed with AD were included in the model training to address class imbalance

This study included 297 participants from the Alzheimer’s Disease Neuroimaging Initiative (ADNI) database who met the following criteria summarized in Fig. 1: (a) baseline diagnosis with MCI or AD, (b) availability of T1-weighted sMRI scan, clinical assessments, and genetic polymorphism data at baseline, (c) follow-up visits exceeding defined durations. We categorized all MCI subjects into progressive MCI (pMCI) or stable MCI (sMCI) based on their progression to AD during follow-up. Participants with reversed diagnostic status and repeated enrollment in the pMCI or sMCI groups were excluded.

The cross-validation set consisted of 238 MCI subjects from ADNI-1 and ADNI-GO/2 cohorts, with a 48-month follow-up. Additionally, 45 subjects with AD were included in the model training to provide insights into pathological changes and address class imbalance. The independent test set comprised 14 MCI subjects with follow-up durations ranging from 36 to 48 months from the ongoing ADNI-3 cohort, serving as external validation for model generalizability.

### Image preprocessing

The acquisition of T1-weighted sMRI scans involved multiple scanners, each with its customized scanning protocols. The initial preprocessing steps were conducted through FreeSurfer (version 7.1.1), including motion correction, intensity normalization, and skull stripping. This yielded images of 256 × 256 × 256 voxels with a spatial resolution of 1 × 1 × 1 mm^3^. We further cropped the images to match the largest skull-stripped brain size of 160 × 176 × 200 voxels, resulting in a 66.43% reduction in the total image volume. To ensure uniformity, image intensities were scaled to a range between 0 and 1 using max–min normalization. The above process is detailed in Additional file 1: Fig. S1.

### Preparation of clinical and genetic features

For each participant, we considered 14 clinical features at baseline, including demographic data (age, sex, education) and cognitive assessments, as listed in Table 1. Sex was encoded as a binary variable. Mean imputation was employed for handling missing data. Subsequently, all variables were normalized using the Min–Max scaler.

**Table 1:** Baseline characteristics

Characteristics	All (n = 252)	Cross-validation set (n = 238)	Independent test set (n = 14)
Age, mean (SD), years	73.2 (7.26)	72.8 (7.13)	79.1 (7.17)
Sex, n (%)			
Female	95 (37.7)	91 (38.2)	4 (28.6)
Male	157 (62.3)	147 (61.8)	10 (71.4)
Education, mean (SD), years	15.9 (2.89)	15.9 (2.87)	15.6 (3.46)
Cognitive test scores			
MMSE, mean (SD)	27.7 (1.80)	27.6 (1.79)	28.6 (1.70)
CDRSB, mean (SD)	1.50 (0.994)	1.50 (0.991)	1.50 (1.07)
ADAS11, mean (SD)	9.61 (4.24)	9.64 (4.25)	8.97 (4.20)
ADAS13, mean (SD)	15.4 (6.66)	15.5 (6.67)	14.0 (6.57)
RAVLT immediate, mean (SD)	35.8 (10.8)	35.7 (10.7)	37.9 (12.7)
RAVLT learning, mean (SD)	4.27 (2.65)	4.29 (2.68)	4.00 (2.11)
RAVLT % forgetting, mean (SD)	57.0 (34.7)	57.2 (34.6)	54.9 (38.1)
mPACCdigit, mean (SD)	− 5.53 (4.31)	− 5.68 (4.26)	− 3.06 (4.64)
mPACCtrailsB, mean (SD)	− 5.19 (4.09)	− 5.34 (4.05)	− 2.61 (4.11)
FAQ, mean (SD)	3.53 (4.51)	3.55 (4.55)	3.14 (3.90)
LDELTOTAL, mean (SD)	6.57 (4.41)	6.36 (4.25)	10.1 (5.70)

*MMSE* Mini-Mental State Examination, *CDRSB* Clinical Dementia Rating Sum of Boxes, *ADAS* Alzheimer’s Disease Assessment Scale, *RAVLT* Rey Auditory Verbal Learning Test, *mPACCdigit* Modified Preclinical Alzheimer Cognitive Composite with Digit, *mPACCtrailsB* Modified Preclinical Alzheimer Cognitive Composite with Trails B, *FAQ* Functional Activity Questionnaire, *LDELTOTAL* Delayed Total Recall

Whole genome sequencing (WGS) data of all participants were genotyped using the Human 610-Quad Bead-Chip. Quality control, encompassing criteria such as genotype quality, deletion rate, minor allele frequency, and Hardy–Weinberg test, was applied to retain 8,326,239 features from 44,535,780 single nucleotide polymorphisms (SNPs). Following data filtering, the genotypes of all SNPs were imputed using Beagle and recoded as the number of alleles. Subsequently, we implemented a two-stage feature selection approach. In the first stage, a knowledge-driven approach selected 1023 AD-related SNPs that had achieved gene-wide significance in the IGAP meta-analysis [28]. In the second stage, a data-driven approach with Lasso regression was performed to identify the most important 49 features. The detailed process is summarized in Additional file 1: Fig. S2.

### Deep learning model architecture

**Fig. 2:**
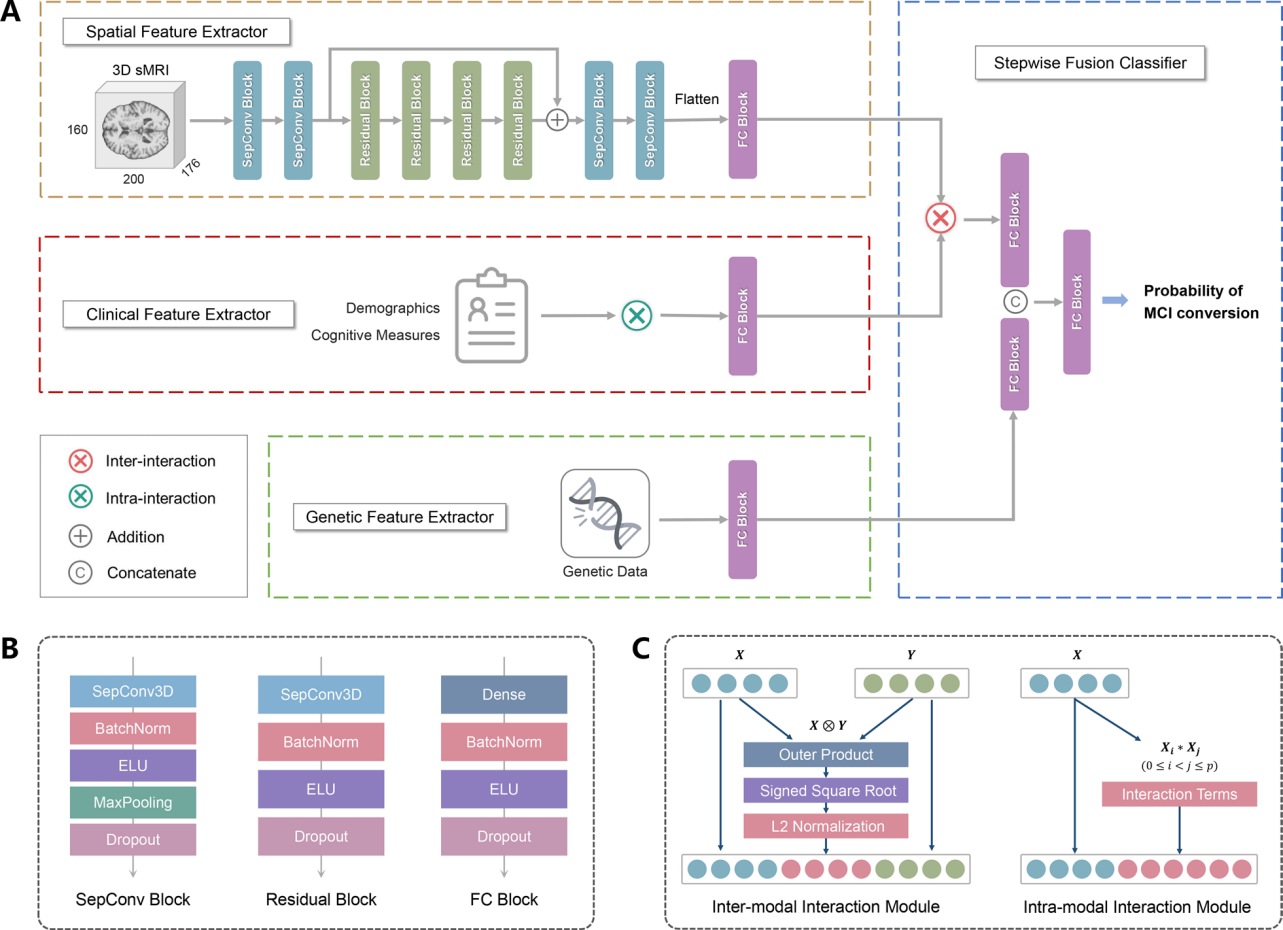
Schematic illustration of the deep learning model architecture. **A** The proposed deep learning model consists of multimodal feature extraction and stepwise fusion classification. **B** Sequential operations within the SepConv block, residual block, and FC block. **C** Inter-modal and intra-modal interaction modules

We proposed the Dual Interaction Stepwise Fusion Classifier (DISFC), a multimodal deep learning model based on 3D sMRI scans, demographic and neuropsychological assessments, and genetic polymorphism data to predict the risk of MCI progression to AD at baseline. The DISFC framework was designed for two steps: multimodal feature extraction and stepwise fusion classification (Fig. 2A). In the multimodal feature extraction step, we employed a parallel three-branch network comprising spatial, clinical, and genetic feature extractors. The network took trimodal data as inputs and produced 8-dimensional abstract features for each modality. In the subsequent stepwise fusion classification step, these abstract representations were gradually fused, starting with the fusion of neuroimaging and clinical high-level features and followed by the concatenation of genetic encoded features. Finally, this process outputted a probability of whether the corresponding MCI patient would convert to AD.

The model backbone consisted of three types of blocks: separable convolution (SepConv) blocks, residual blocks, and fully connected (FC) blocks (Fig. 2B). To prevent overfitting, we implemented batch normalization, dropout, and L2 regularization in each block. In the spatial feature extractor of the DISFC model, SepConv blocks replaced traditional 3D convolution with separable convolution to reduce the number of trainable parameters. A shortcut connection was applied to the group of four residual blocks to improve gradient propagation.

Most importantly, our DISFC model introduced two interaction modules: the intra-modal interaction module and the inter-modal interaction module (Fig. 2C). The dual interaction modules played a pivotal role in guiding the model to learn meaningful combinations. The intra-modal interaction module was applied to the clinical feature extractor of the DISFC model to integrate clinical variables and their second-order interaction terms. Meanwhile, the inter-modal interaction module was embedded into the stepwise fusion process, explicitly modeling the pairwise interactions between neuroimaging and clinical information using outer product.

### Model development and evaluation

Sigmoid activation and binary cross-entropy loss were applied to the output layers of the three feature extractors and the stepwise fusion classifier to supervise the learning of our model. The binary cross-entropy loss is defined as follows:

$${\mathcal{L}}\left( {y,\hat{y}} \right) = - \frac{1}{N}\sum\limits_{i = 1}^{N} {\left[ {y_{i} \cdot \log \left( {\hat{y}_{i} } \right) + \left( {1 - y_{i} } \right) \cdot \log \left( {1 - \hat{y}_{i} } \right)} \right]} ,$$

where $N$ is the batch size, $y_{i}$ represents the ground truth for sample $i$, and $\hat{y}_{i}$ is the conversion probability of sample $i$ predicted by our model.

Based on the described design, our complete loss function ${\mathcal{L}}$ for the MCI conversion prediction task is as follows:

$${\mathcal{L}} = \alpha {\mathcal{L}}_{fusion} + \beta_{1} {\mathcal{L}}_{mri} + \beta_{2} {\mathcal{L}}_{clin} + \beta_{3} {\mathcal{L}}_{snp} ,$$

where ${\mathcal{L}}_{fusion}$ denotes the classification loss for the fusion subnetwork output, and ${\mathcal{L}}_{mri}$, ${\mathcal{L}}_{clin}$, and ${\mathcal{L}}_{snp}$ represent the corresponding losses for three feature extractors. $\alpha$ and $\beta_{i}$ $\left( {i = 1, 2, 3} \right)$ are the hyperparameters for balancing losses, set to 1.0, 1.5, 0.5, and 0.5 in our experiments.

All experiments were conducted using Keras with TensorFlow backend on NVIDIA Tesla V100 GPUs. During model development, we performed stratified tenfold cross-validation to ensure each patient was tested once. Data augmentation was applied to augment training data, including mirroring, rotation, shifting, and scaling transformations for sMRI, as well as slight random perturbations for clinical and genetic inputs. The model was trained for 50 epochs with a batch size of 6. We utilized the Adam optimizer with a learning rate that initially warmed up to 0.001 in 15 epochs and then exponentially decayed. The best-performing model, determined by validation performance, was evaluated on the independent test set to assess its generalizability.

The performance of the DISFC model on the cross-validation and the independent test sets was evaluated using metrics including the area under the receiver operating characteristic curve (AUC), accuracy, sensitivity, specificity, and F1 score. Notably, we calculated the average results across folds as the overall performance of the tenfold cross-validation.

### Statistical analysis

All statistical analyses were implemented using R software (version 4.1.2). We compared the performance of the validation and independent test sets using the Fisher exact test for accuracy, sensitivity, specificity, and the Delong test for AUC. Similarly, we applied the Fisher exact test to evaluate differences in accuracy, sensitivity, and specificity between subgroups from different centers and scanners. The Wilcoxon signed rank test with continuity correction was employed to assess the improvement by interaction modules and multimodality. For comparisons between our model and previous methods, we utilized the same test for AUC and conducted the one-sample proportions test for accuracy, sensitivity, and specificity. The 95% confidence intervals (CIs) were calculated by the Clopper–Pearson method for accuracy, sensitivity, specificity, and 2000 stratified bootstrap replicates for AUC. The p-values less than 0.05 were considered statistically significant.

## Results

### Baseline characteristics

This study included 252 patients diagnosed with MCI at enrollment, comprising 157 (62.3%) men and 95 (37.7%) women. The mean age across the datasets was 73.2 years, with a standard deviation (SD) of 7.26 years. Of the 252 MCI patients, 94 (37.3%) progressed to AD, and 158 (62.7%) remained stable during follow-up. The baseline characteristics for the cross-validation and the independent test sets are outlined in Table 1.

### Performance of the deep learning model

**Fig. 3:**
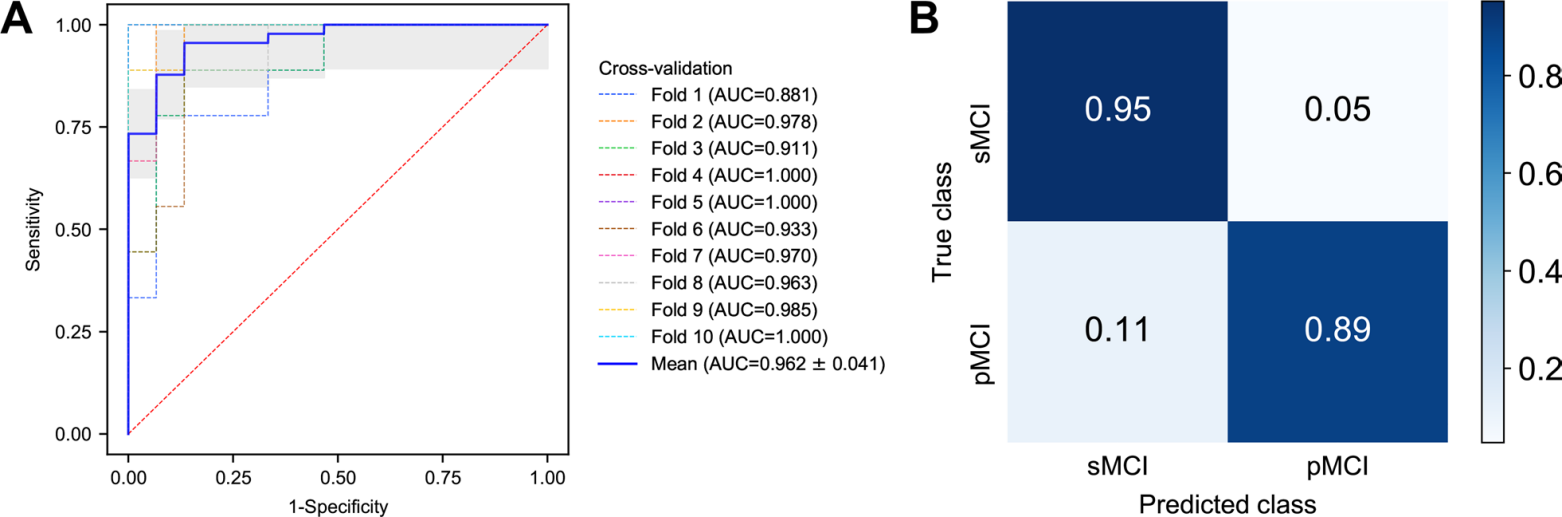
Performance of the deep learning model on the cross-validation set. **A** Receiver operating characteristic (ROC) curves of tenfold cross-validation. The mean ROC curve with an AUC of 0.962 was obtained by interpolating the ROC curves for tenfolds. Gray shading indicates ± 1 SD of the mean curve. **B** Confusion matrix of the proposed model on the cross-validation set

On the cross-validation set, the DISFC model achieved a mean (SD) of 0.962 (0.041) for AUC for predicting MCI conversion over 4 years (Fig. 3A). The average (SD) accuracy, sensitivity and specificity were 92.92% (4.41%), 88.89% (9.07%) and 95.33% (4.50%), respectively (Fig. 3B). On the independent test set, DISFC demonstrated good generalizability with an AUC of 0.939 (95% CI 0.796–1.000) and accuracy of 92.86% (95% CI 66.13–99.82%). There was no significant difference in predictive performance between cross-validation and independent test (AUC, *P* = 0.55; accuracy, *P* = 1.00; sensitivity, *P* = 0.46; specificity, *P* = 1.00). The DISFC model correctly classified all sMCI cases in the independent test set, with only one misclassification observed among the three pMCI cases. The longitudinal analysis of this specific pMCI case revealed that, despite an initial misclassification at baseline (4 years before conversion), our DISFC model accurately predicted the conversion from MCI to AD 2 years in advance. Further validation of model generalization found that the DISFC model exhibited consistent generalization performance on the enlarged independent test set (Additional file 1: Extended validation for model generalization capability).

### Improving prediction accuracy through interaction effects and multimodality

**Fig. 4:**
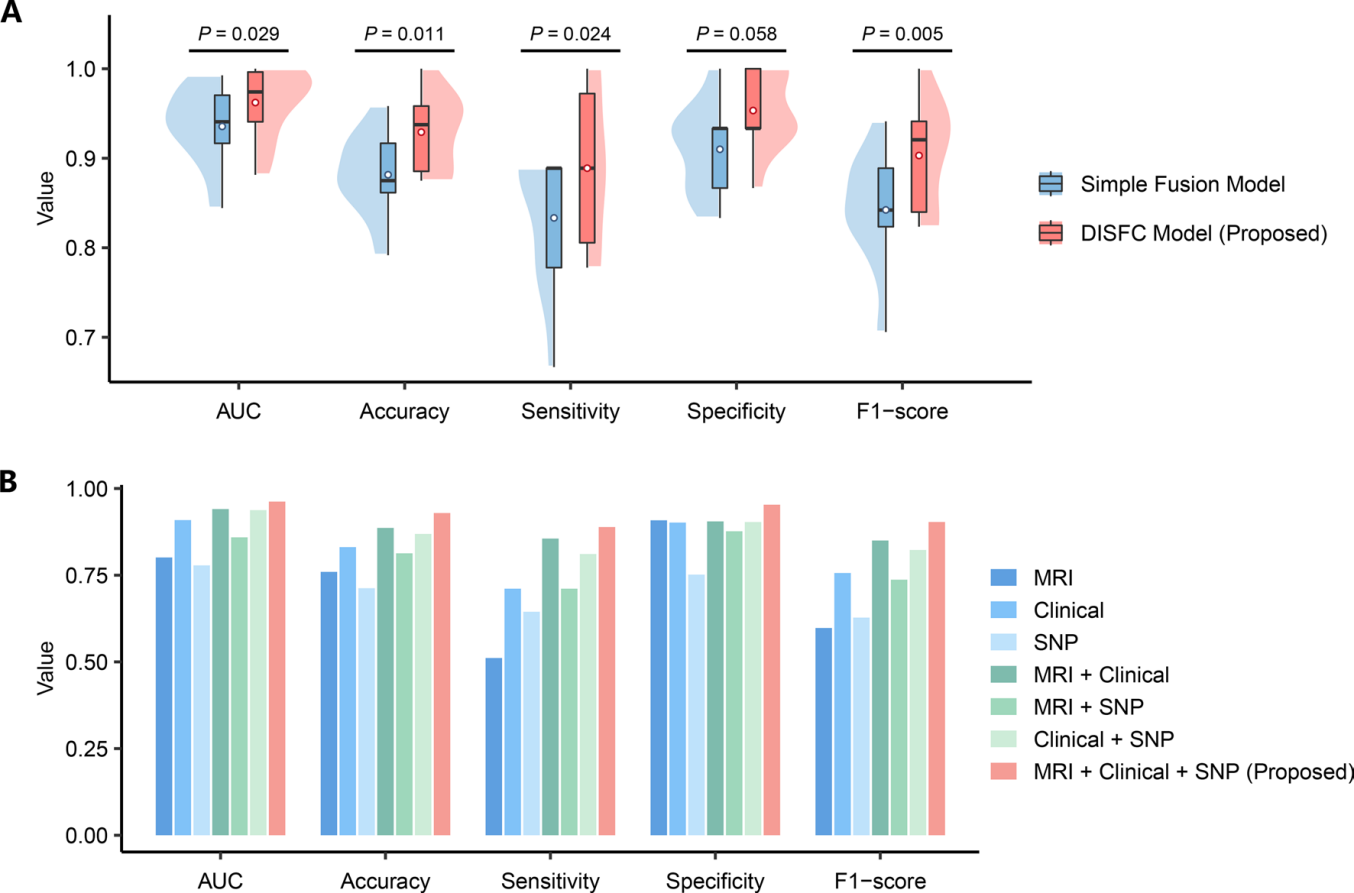
Comparison of model performance with and without interaction effects and multimodality. **A** Effectiveness evaluation of the dual interaction modules. The performance of our DISFC model was compared with the simple fusion benchmark model without intra-modal and inter-modal interaction modules. The box plot illustrates the 25th percentile (upper box limit), median (horizontal centerline), and 75th percentile (lower box limit). The upper whisker, lower whisker, and hollow circle symbol indicate the maximum, minimum, and mean values of a given model for each metric, respectively. The shaded area on one side around each box represents the probability density. **B** Performance comparison of the models based on different modality combinations. The unimodal, bimodal, and trimodal models were cross-validated with identical settings. Each bar represents the mean value across folds for each metric

To evaluate the impact of incorporating interaction effects, we constructed a simple fusion model as a benchmark. This model shared a similar architecture with DISFC, except for the absence of intra-modal and inter-modal interaction modules, as shown in Additional file 1: Fig. S3. Under the same training settings, the DISFC model showed a significant increase in AUC, accuracy, sensitivity, and F1 score compared to the simple fusion benchmark model (Fig. 4A). The AUC improved by 0.027 (*P* = 0.03), accuracy by 4.76% (*P* = 0.01), sensitivity by 5.56% (*P* = 0.02), and F1 score by 6.11% (*P* = 0.005).

To estimate the influence of multimodality on predictive performance, we compared models based on different combinations of modalities. As shown in Fig. 4B, the DISFC model using trimodal data achieved the best performance. We also observed that bimodal models outperformed unimodal models overall. The trimodal DISFC model was superior to the top-performing bimodal model using MRI and clinical data with a significant increase of 0.022 (*P* = 0.048) in AUC, 4.29% (*P* = 0.03) in accuracy, and 4.83% (*P* = 0.02) in specificity.

### Comparisons with existing methods

**Fig. 5:**
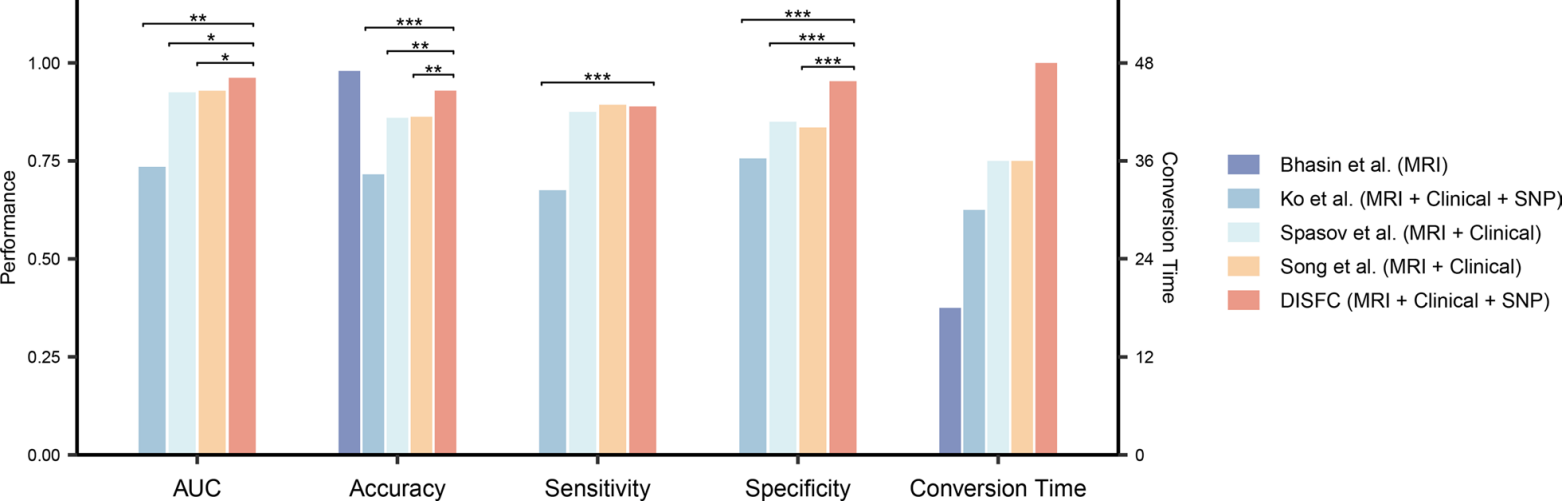
Comparison of the performance of our proposed model with other state-of-the-art models using the ADNI database. P-values were calculated to compare the performance of previous models with our proposed DISFC model. *P < 0.05; **P < 0.01; ***P < 0.001

The DISFC model exhibited superior predictive performance and an extended prediction horizon for MCI conversion compared to several state-of-the-art methods using the ADNI database (Fig. 5). Compared with Spasov et al. [24] and Song et al. [25] using joint bimodal information, DISFC showed higher AUC (0.962 vs. 0.925, *P* = 0.03; 0.962 vs. 0.929, *P* = 0.04), increased accuracy (92.92% vs. 86.00%, *P* = 0.003; 92.92% vs. 86.27%, *P* = 0.005), greater specificity (95.33% vs. 85.00%, *P* < 0.001; 95.33% vs. 83.53%, *P* < 0.001), and comparable sensitivity (88.89% vs. 87.50%, *P* = 0.81; 88.89% vs. 89.33%, *P* > 0.99). A plausible explanation for the observed improvements is the incorporation of multimodal data and interaction modules in our model. However, despite using trimodal data like ours, Ko et al. [29] achieved much lower AUC (0.735 vs. 0.962, *P* = 0.006), accuracy (71.59% vs. 92.92%, *P* < 0.001), sensitivity (67.53% vs. 88.89%, *P* < 0.001), and specificity (75.64% vs. 95.33%, *P* < 0.001) than DISFC. This is because their model primarily emphasized inter-modal interaction while overlooking the rich information that each modality can independently provide. Bhasin et al. [10] obtained higher accuracy but in short-term MCI conversion prediction within 18 months. Their study selected images from scanners with the same settings and required additional gray matter segmentation preprocessing.

### Model robustness to disease-independent variability

**Fig. 6:**
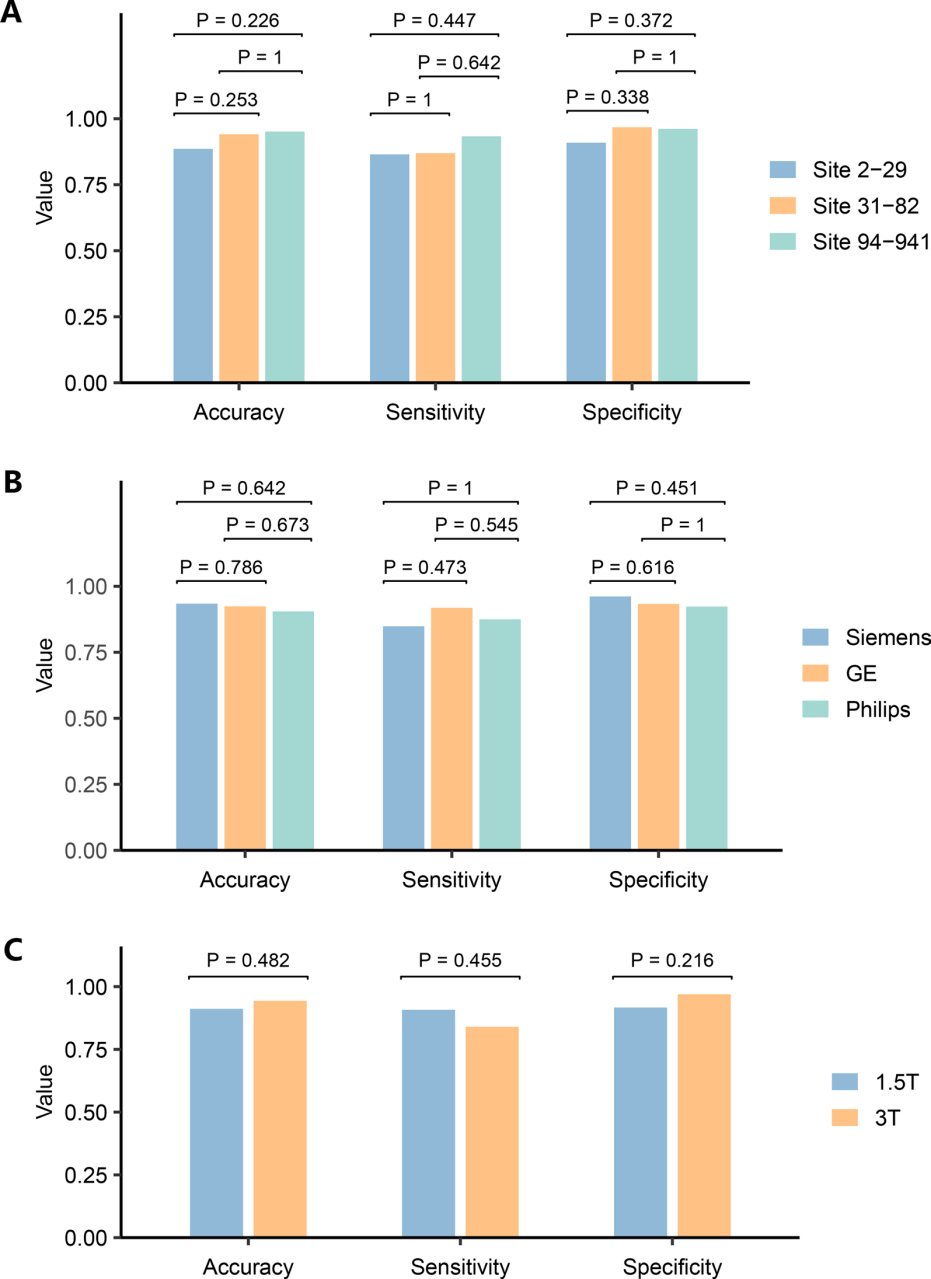
Robustness evaluation across different centers and scanners. **A** Model performance comparison between clinical sites. **B** Model performance comparison between scanner manufacturers. **C** Model performance comparison between scanner magnetic field strengths

To access the robustness of the DISFC model, we compared its predictive performance across different clinical sites, scanner manufacturers, scanner magnetic field strengths and training set sizes. The cross-validation set comprised participants from 52 clinical sites, where sMRI scans were acquired using scanners with two different magnetic field strengths (1.5 T and 3 T) from three distinct manufacturers (Siemens, GE, and Philips). First, we divided the cross-validation set into three groups based on clinical sites. The DISFC model exhibited consistent accuracy, sensitivity, and specificity across these subgroups, indicating its robustness to data from various clinical sites (Fig. 6A). For scanners, there were no significant differences in accuracy, sensitivity, and specificity across different manufacturers and magnetic field strengths, suggesting that the DISFC model had good tolerance for scanning variability (Fig. 6B, C). Furthermore, our model exhibited adaptability to reduced training data regarding predictive performance (Additional file 1: Fig. S4).

### Ablation studies on basic model architecture

**Fig. 7:**
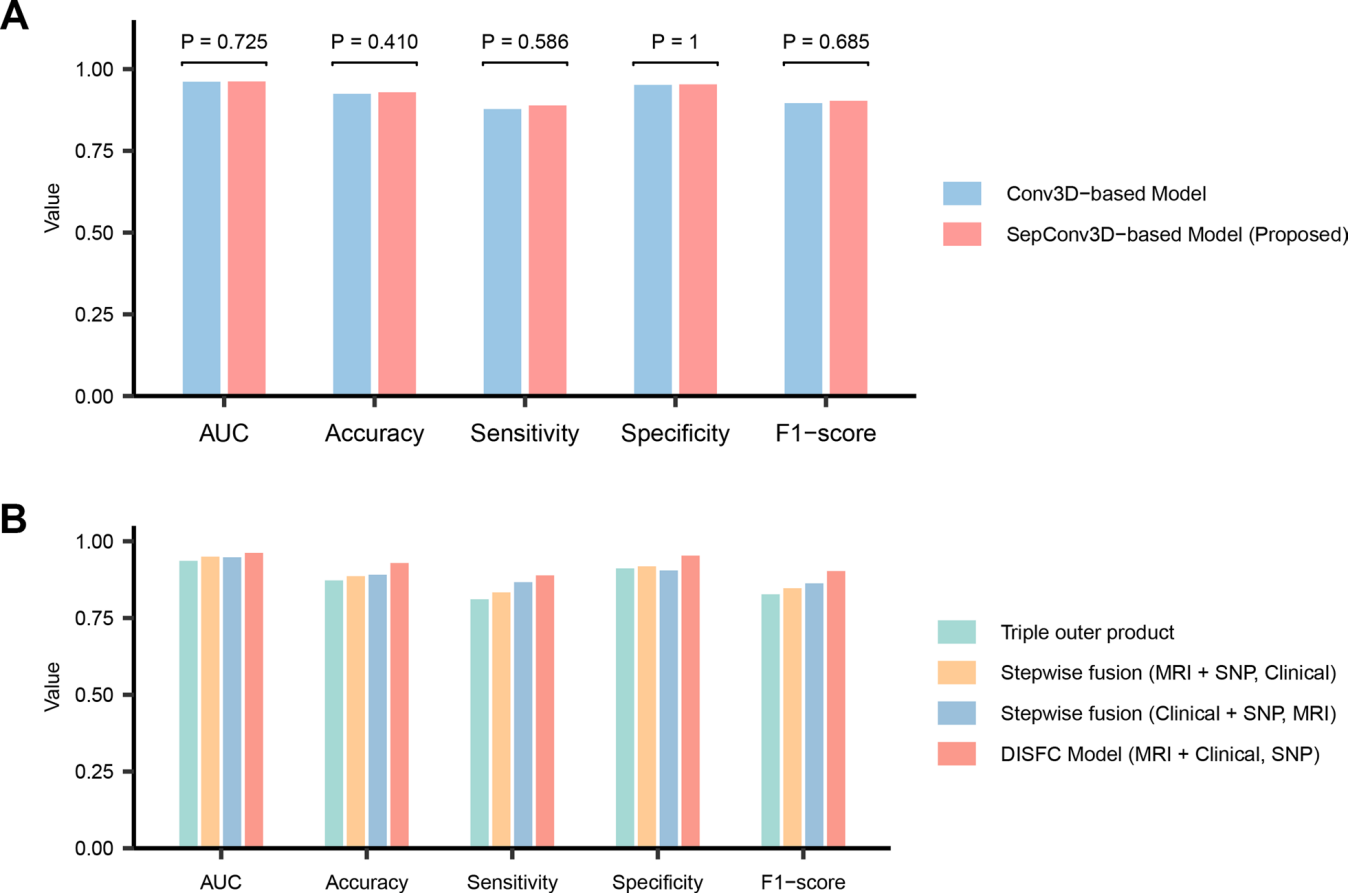
Ablation studies on basic model architecture. **A** Performance comparison of the models using classical convolution and separable convolution. The models employing classical 3D convolution (Conv3D-based) and separable 3D convolution (SepConv3D-based) were cross-validated with identical settings. Each bar in the chart represents the mean value across folds for respective metrics. **B** Performance comparison of models based on different fusion schemes. The models based on triple outer product fusion and stepwise fusion were cross-validated under the same settings. Each bar in the chart represents the mean value across folds for respective metrics

We conducted extensive ablation experiments to indicate the rationale behind the basic architecture of our model. To explore the impact of separable convolution, we extended the DISFC model with a benchmark model that replaced separable convolution with classical convolution in the spatial feature extractor. As illustrated in Fig. 7A, our DISFC model based on separable convolution was comparable to the classical convolution-based model across metrics including AUC (*P* = 0.73), accuracy (*P* = 0.41), sensitivity (*P* = 0.59), specificity (*P* = 1.00), and F1 score (*P* = 0.69). This suggests that our proposed model is lightweight without sacrificing predictive performance. Similar findings emerged from comparative experiments with different backbones in the spatial feature extractor (Additional file 1: Fig. S5). Furthermore, we compared models using different fusion schemes, including triple outer product fusion and three stepwise fusion approaches, as listed in Fig. 7B. The experimental results demonstrated superior performance of our DISFC model over models with alternative fusion schemes. Details on selecting optimal residual connection number and excluding the genetic intra-modal interaction module can be found in Additional file 1: Figs. S6 and S7.

### Interpretation and visualization for multimodal contribution

**Fig. 8:**
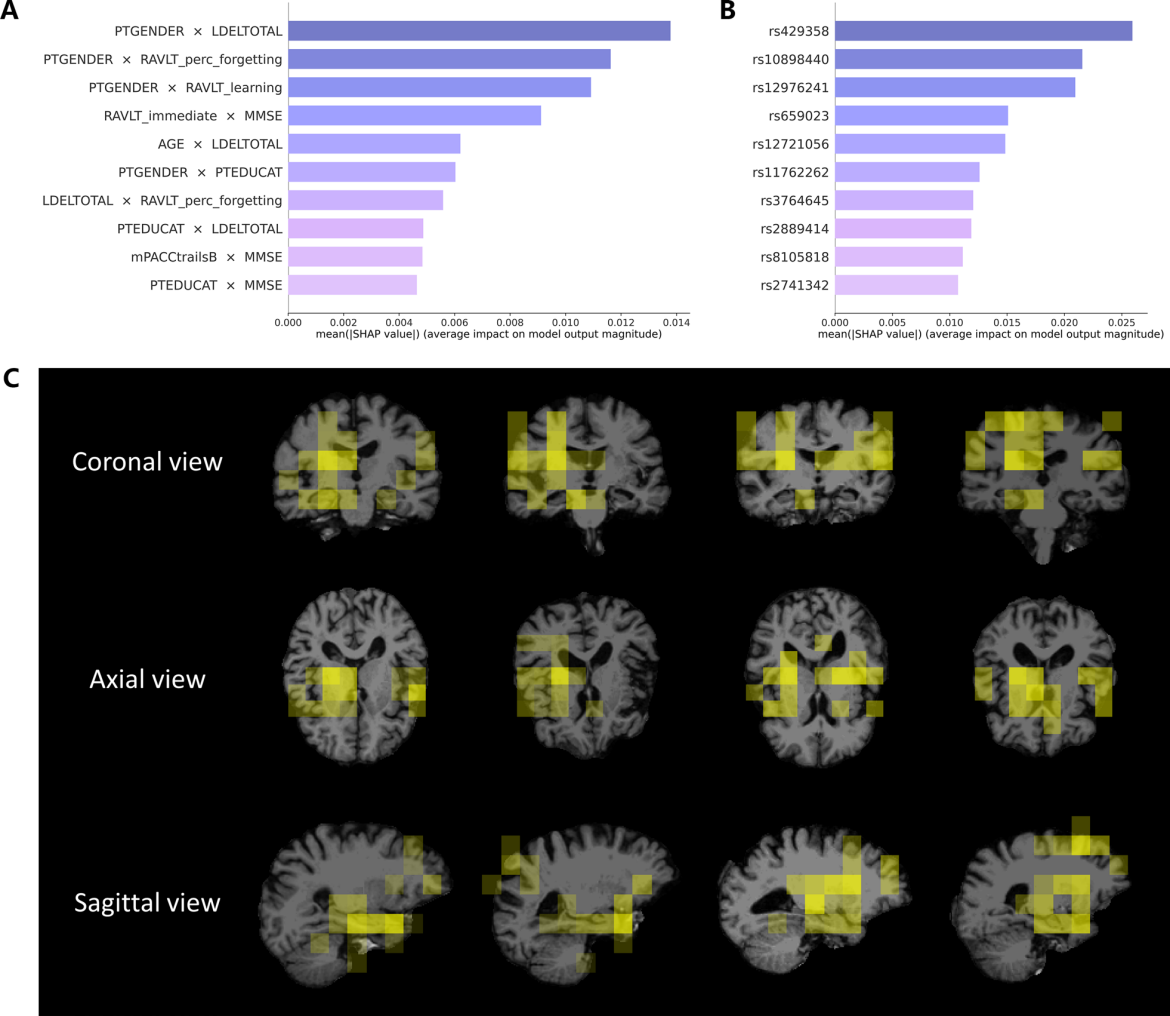
Visualization of the importance of multimodal features. **A** The top 10 features of most interest to the clinical feature extractor in our model. **B** The top 10 features of most interest to the genetic feature extractor in our model. **C** The top 15 brain regions of most interest to the spatial feature extractor in our model, depicted in coronal, axial, and sagittal views for four representative pMCI cases. The color transparency represents the level of importance of the brain region

We utilized the Shapley Additive Explanation (SHAP) method [30] to uncover how multimodal biomarkers contribute to the predictive capability of our DISFC model. The clinical features of greatest importance were exclusively interaction terms, indicating that the clinical feature extractor of the DISFC model primarily depended on intra-modal interaction (Fig. 8A). As illustrated in Fig. 8B, the genetic feature extractor of the DISFC model highlighted several SNPs, including rs429358, rs10898440, rs12721056, rs11762262, rs3764645, rs2889414, rs8105818, and rs2741342. These SNPs can be mapped to AD-related genes, such as APOE [31], PICALM [32], APOC1 [33], EPHA1 [34], ABCA7 [35], CBLC [36], BLOC1S3 [37], and CHRNA2 [38]. Figure 8C depicts that the DISFC model also prioritized neuroimaging biomarkers in regions such as the hippocampus, amygdala, thalamus, lateral ventricle, cortical sulci, and gyri, all of which are associated with AD [39, 40]. We also conducted the interpretability analysis on the importance of the features fused from three modalities. The aggregated SHAP values for imaging, clinical, genetic, and imaging-clinical interaction terms were 10.30, 58.51, 50.05, and 21.84, respectively.

## Discussion

In this study, we developed an interpretable DNN model called DISFC to predict the long-term progression from MCI to AD. The model achieved impressive performance with AUCs of 0.962 in the cross-validation set and 0.939 in the independent test set. By incorporating interaction effects and multimodal data in our model, we observed significant accuracy improvements of 4.76% and 4.29%, respectively. Moreover, our model outperformed the state-of-the-art approaches in prediction accuracy and horizon, while demonstrating excellent robustness when applied to multi-center multi-scanner data from the cross-validation set.

Although recent studies have dedicated much effort to MCI conversion prediction, there is still room to enhance their performance and practicality. In contrast to previous works, our DISFC model improves the architectural design of DNN models in two ways. Firstly, it utilizes trimodal features from imaging, clinical, and genetic data to provide insight into disease progression from complementary perspectives. Secondly, it introduces intra-modal and inter-modal interaction modules to extract complex relationships within and across modalities. Building upon these improvements, the DISFC model attained more precise and longer-term predictions for MCI conversion than existing methods.

Predicting MCI conversion can be viewed as a challenging fine-grained classification task, characterized by slight inter-class differences and high intra-class variance. In our study, incorporating interaction effects enhanced the representation power of the DISFC model to distinguish between pMCI and sMCI, especially when high-quality medical data for training were limited. Our results demonstrated that traditional concatenation-based fusion algorithms fell short of fully capturing complex interactions. On the other hand, the DISFC framework provided the opportunity to explicitly model intricate interactions among diverse features, thanks to the presence of intra-modal and inter-modal interaction modules. The dual interaction modules can effectively capture subtle differences within and across modalities, making our model more sensitive to finely differentiated pathological changes.

Model generalization is critical for the clinical applicability of computer-aided diagnosis. However, the individual heterogeneity among MCI patients and the variability in data acquisition protocols constrain the generalizability of current MCI conversion prediction algorithms to real-world clinical data, thereby introducing additional challenges for their practical implementation. In our study, due to the support of multimodality and interaction modules, the DISFC model had good adaptability for unknown data and multiple disease-independent factors. The DISFC model achieved comparable performance on internal validation and independent test sets. Furthermore, the dataset we used in this study encompassed various clinical centers, imaging device manufacturers, and magnetic field strengths. The positive outcomes across diverse data scenarios confirm the robustness of the DISFC model and show its potential for widespread clinical applications.

The reservations of clinicians to embrace artificial intelligence in healthcare often stem from concerns related to the black-box problem [41]. To address this issue, we conducted a post-hoc interpretation of the DISFC model to investigate the correlation between its underlying mechanism and medical consensus in imaging, cognition, and genetics. The visualization results revealed that the DISFC model identified patterns of brain atrophy from the imaging input, including hippocampal atrophy and ventricular enlargement. These highlighted biomarkers are also acknowledged as valid indicators of neurodegeneration in AD. Similarly, the genetic feature extractor of the DISFC model focused on SNP biomarkers situated in regions of previously reported AD-related genes. Besides, the DISFC model prioritized clinical features that involved interactions within demographic and neuropsychological characteristics. Moreover, the imaging-clinical interactions offered additional assistance in prediction beyond the information provided by the two modalities themselves. These not only reflect decision-making process in clinical practice but also underscore the imperative incorporation of the intra-modal and inter-modal interaction modules into our model. The findings above suggest that our model is built upon prior knowledge of dementia neuroscience. Therefore, it can offer more reliable predictions for computer-aided diagnosis.

This study has some limitations. Firstly, despite utilizing data collected from various centers and devices, this study exclusively included subjects from a single institution, ADNI. Participants in the ADNI cohorts were predominantly well-educated and of white ethnicity, which may affect data representativeness. Another limitation is the small sample size, which is a consequence of the inclusion criteria requiring multimodal completeness and sufficient follow-up duration. To address this issue, we employed data augmentation to enrich the available training data and utilized separable convolution to extract 3D spatial features with reduced parameters. Batch normalization, dropout, and regularization were also built into our model to mitigate overfitting. In future studies, our model should undergo further evaluation on large-scale multi-institutional datasets.

## Conclusions

In this study, we proposed a deep learning model for the long-term prediction of MCI-to-AD progression. Our model achieved superior performance compared to the state-of-the-art studies, demonstrated generalizability to unseen data, and showed robustness to inter-center and inter-scanner variability. The findings emphasize the immense potential of integrating interactive effects and multimodality into deep learning frameworks for the precise and cost-effective prediction of MCI conversion at the individual level, which is expected to advance early diagnosis of AD.

### Supplementary Information

**Additional file 1: Figure S1.** sMRI preprocessing workflow. **Figure S2.** Genetic feature filtering and selection workflow. **Figure S3.** Schematic illustration of the simple fusion benchmark model. **Figure S4.** Performance trends for models with different training set sizes. **Figure S5.** Performance comparison of models based on different spatial feature extractor backbones. **Figure S6.** Performance comparison of models using different residual connection methods. **Figure S7.** Performance comparison of the models with and without genetic intra-modal interaction.

## Data Availability

The datasets generated and/or analyzed during the current study are available in the Alzheimer’s disease Neuroimaging Initiative repository, http://adni.loni.usc.edu.

## Abbreviations

AD: Alzheimer’s disease
ADAS: Alzheimer’s Disease Assessment Scale
ADNI: Alzheimer’s Disease Neuroimaging Initiative
AUC: Area under the receiver operating characteristic curve
CDRSB: Clinical Dementia Rating Sum of Boxes
CI: Confidence interval
DISFC: Dual Interaction Stepwise Fusion Classifier
DNN: Deep neural network
FAQ: Functional Activity Questionnaire
FC: Fully connected
GWAS: Genome-wide association studies
LDELTOTAL: Delayed Total Recall
MCI: Mild cognitive impairment
MMSE: Mini-Mental State Examination
mPACCdigit: Modified Preclinical Alzheimer Cognitive Composite with Digit
mPACCtrailsB: Modified Preclinical Alzheimer Cognitive Composite with Trails B
pMCI: Progressive mild cognitive impairment
RAVLT: Rey Auditory Verbal Learning Test
ROC: Receiver operating characteristic
SD: Standard deviation
SepConv: Separable convolution
SHAP: Shapley Additive Explanation
sMCI: Stable mild cognitive impairment
sMRI: Structural magnetic resonance imaging
SNP: Single nucleotide polymorphism
WGS: Whole genome sequencing
