# Supplementary material for: Predicting long-term progression of Alzheimer’s disease using a multimodal deep learning model incorporating interaction effects
Source: J Transl Med. 2024 Mar 11;22:265. doi: 10.1186/s12967-024-05025-w (PMC10926590; doi:10.1186/s12967-024-05025-w)
Supplement: Supplementary file 1 — Additional file 1: Figure S1. sMRI preprocessing workflow. Figure S2. Genetic feature filtering and selection workflow. Figure S3. Schematic illustration of the simple fusion benchmark model. Figure S4. Performance trends for models with different training set sizes. Figure S5. Performance comparison of models based on different spatial feature extractor backbones. Figure S6. Performance comparison of models using different residual connection methods. Figure S7. Performance comparison of the models with and without genetic intra-modal interaction. [file 12967_2024_5025_MOESM1_ESM.docx]

**Additional file for**

**Predicting long-term progression of Alzheimer’s disease using a multimodal deep learning model incorporating interaction effects**

Yifan Wang^1, 2^, Ruitian Gao^1, 2^, Ting Wei^1, 2^, Luke Johnston^3^, Xin Yuan^1, 2^, Yue Zhang^1, 2^, Zhangsheng Yu^1, 2, 3, 4, *^, for the Alzheimer’s Disease Neuroimaging Initiative

1. Department of Bioinformatics and Biostatistics, School of Life Sciences and Biotechnology, Shanghai Jiao Tong University, Shanghai, China;

2. SJTU-Yale Joint Center for Biostatistics and Data Science, Shanghai Jiao Tong University, Shanghai, China;

3. School of Mathematical Sciences, Shanghai Jiao Tong University, Shanghai, China;

4. Clinical Research Institute, Shanghai Jiao Tong University School of Medicine, Shanghai, China.

* Corresponding author:

Zhangsheng Yu, Ph.D. Department of Bioinformatics and Biostatistics, School of Life Sciences and Biotechnology, Shanghai Jiao Tong University, 800 Dongchuan Road, Minhang District, Shanghai, 200240, China. Email: yuzhangsheng@sjtu.edu.cn

**Extended validation for model generalization capability**

To further assess the generalization capability of our proposed DISFC model, we stratified 47 MCI subjects (20% of the original cross-validation set comprising ADNI-1 and ADNI-2) from ADNI-2, including 18 pMCI and 29 sMCI. These subjects, along with 14 MCI subjects from ADNI-3, formed a new independent test set of 61 MCI subjects in total.

On the new cross-validation set consisting of ADNI-1 and part of ADNI-2, our DISFC model achieved a mean (SD) AUC of 0.954 (0.048), accuracy of 90.34% (5.70%), sensitivity of 87.14% (10.54%), specificity of 92.17% (7.82%), and F1 score of 87.14% (7.30%). On the new independent test set, our model achieved an AUC of 0.967 (95% CI, 0.925-1.000), accuracy of 88.52% (95% CI, 77.78%-95.26%), sensitivity of 85.71% (95% CI, 63.66%-96.95%), and specificity of 90.00% (95% CI, 76.34%-97.21%). There was no significant difference in predictive performance between cross-validation and independent test (AUC, *P* = 0.12; accuracy, *P* = 0.19; sensitivity, *P* = 0.55; specificity, *P* = 0.56).


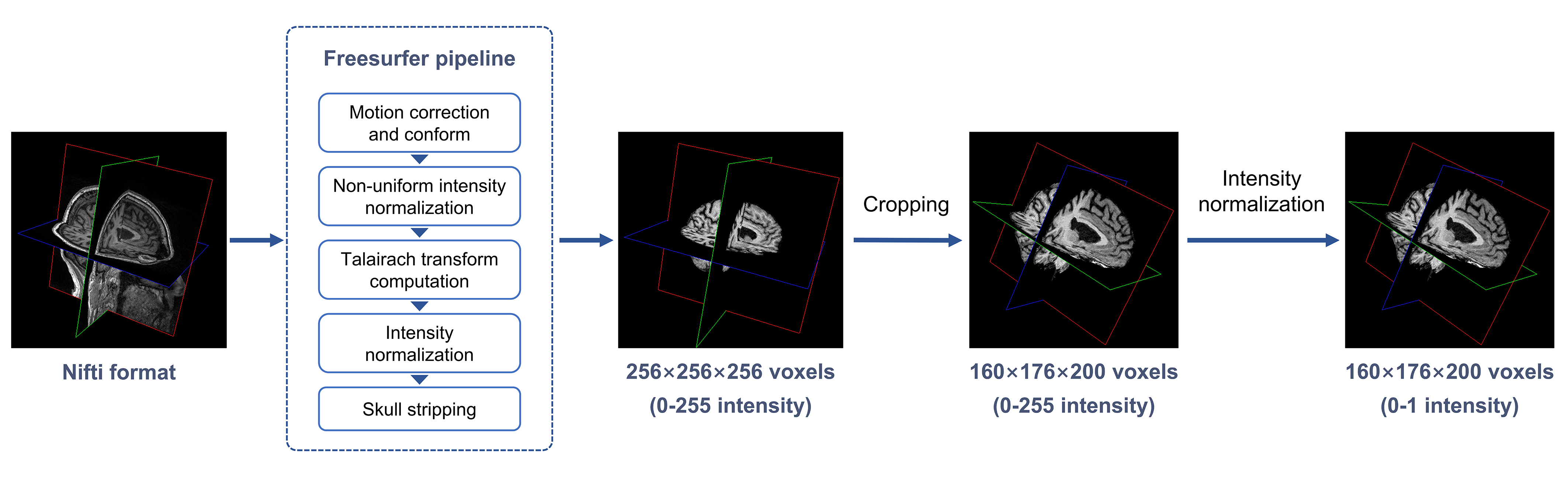


**Figure S1. sMRI preprocessing workflow.**

The initial Nifti format sMRI scans underwent preliminary preprocessing using the Freesurfer software standard pipeline, resulting in images of 256 × 256 × 256 voxels. Subsequently, we cropped the images to 160 × 176 × 200 voxels to retain the largest skull-stripped brain size. Finally, image intensities were normalized to a range between 0 and 1 for each subject.


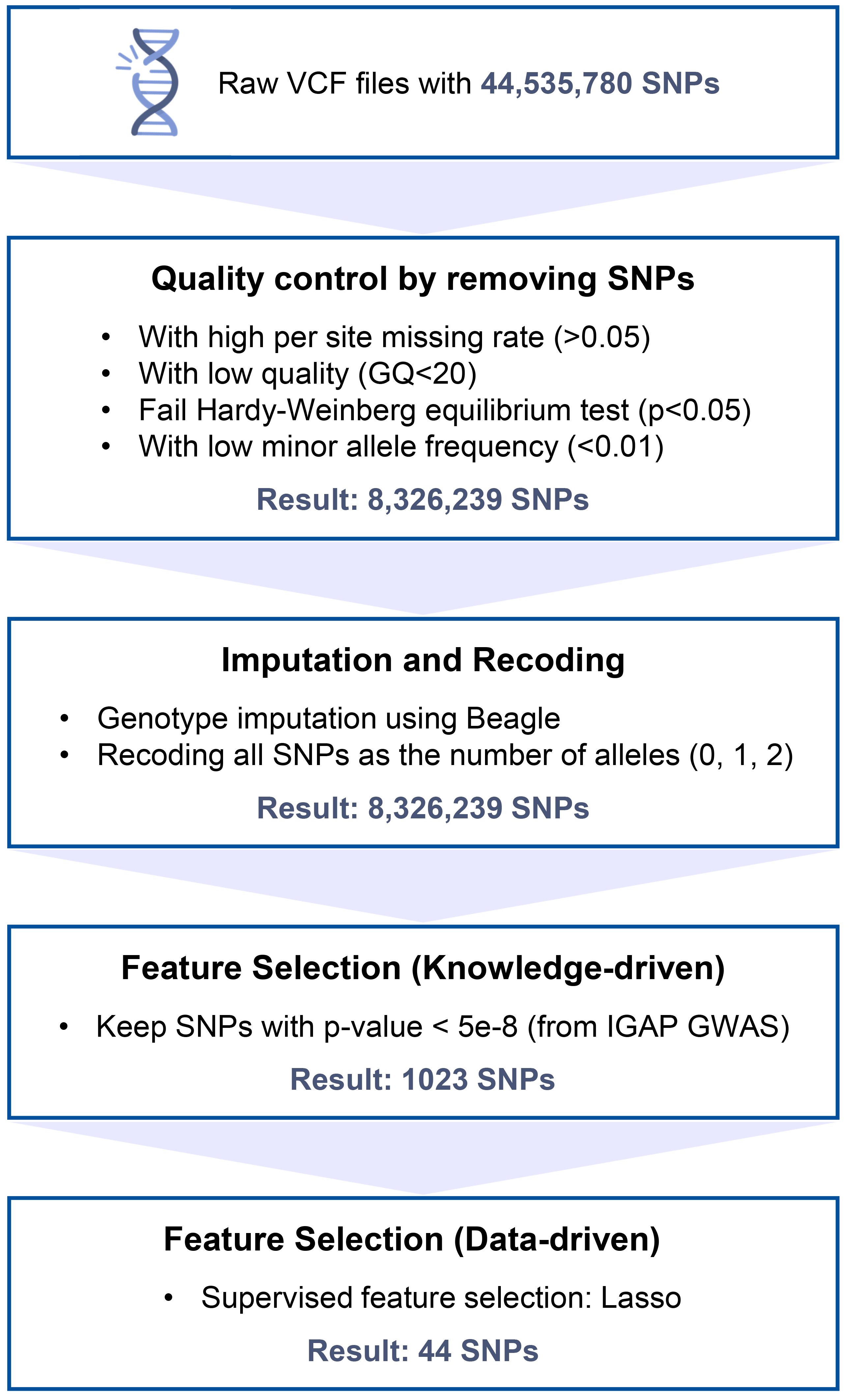


**Figure S2. Genetic feature filtering and selection workflow.**

The original WGS data in VCF format contained 44,535,780 SNPs. We applied quality control criteria, including deletion rate, genotype quality, Hardy Weinberg test, and minor allele frequency, resulting in the selection of 8,326,239 SNPs. Subsequently, we performed imputation and recoding to create the genotype matrix. Knowledge-driven feature selection was then employed to retain 1023 AD-related SNPs identified by the IGAP study. Finally, we used Lasso for data-driven feature selection, ultimately retaining 44 SNPs for downstream analysis.


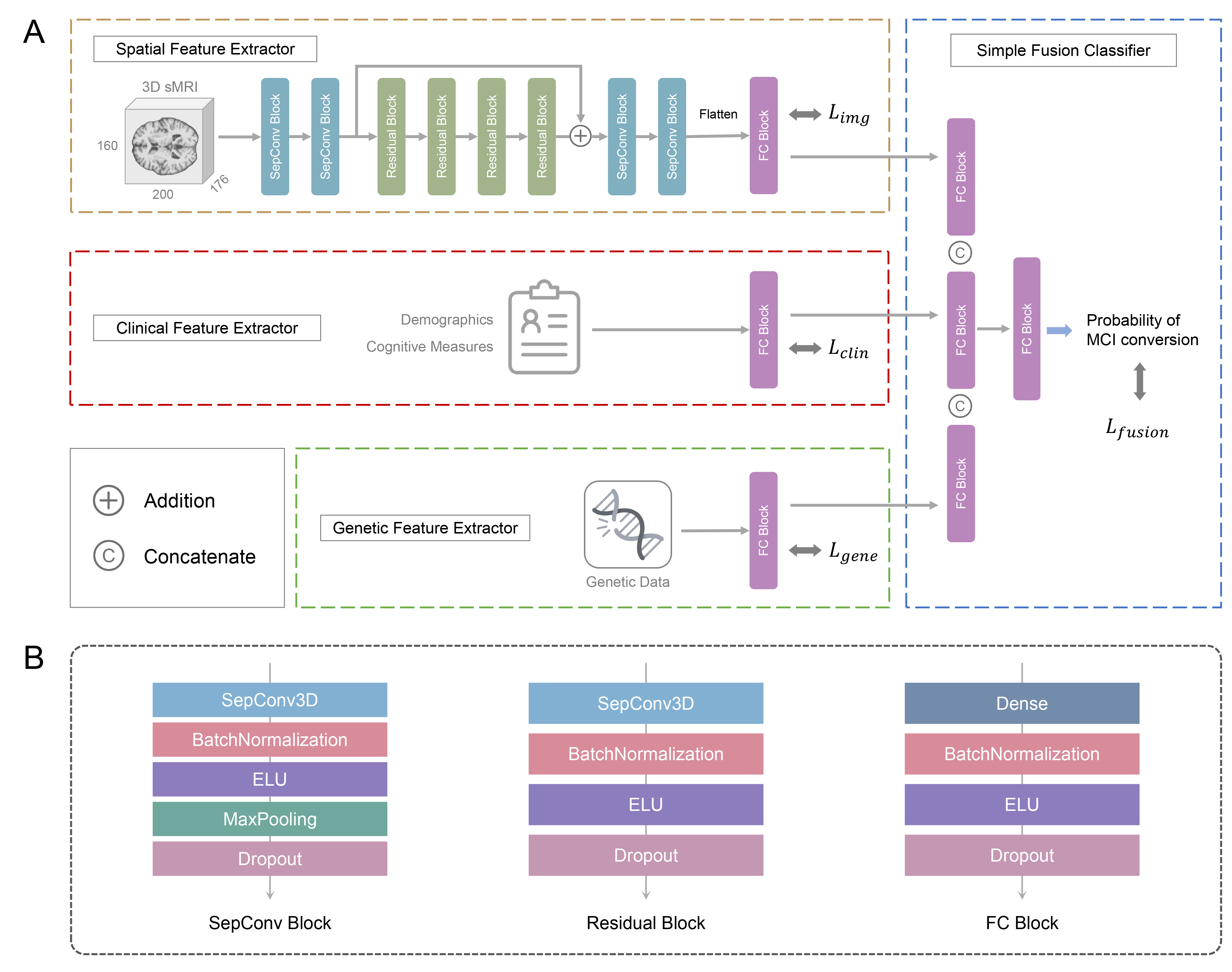


**Figure S3. Schematic illustration of the simple fusion benchmark model.**

(A) The simple fusion model consists of multimodal feature extraction and simple fusion classification. (B) Sequential operations within the separable convolution (SepConv) block, residual block, and fully connected (FC) block. SepConv3D, separable 3D convolution; BatchNorm, batch normalization; ELU, exponential linear unit.


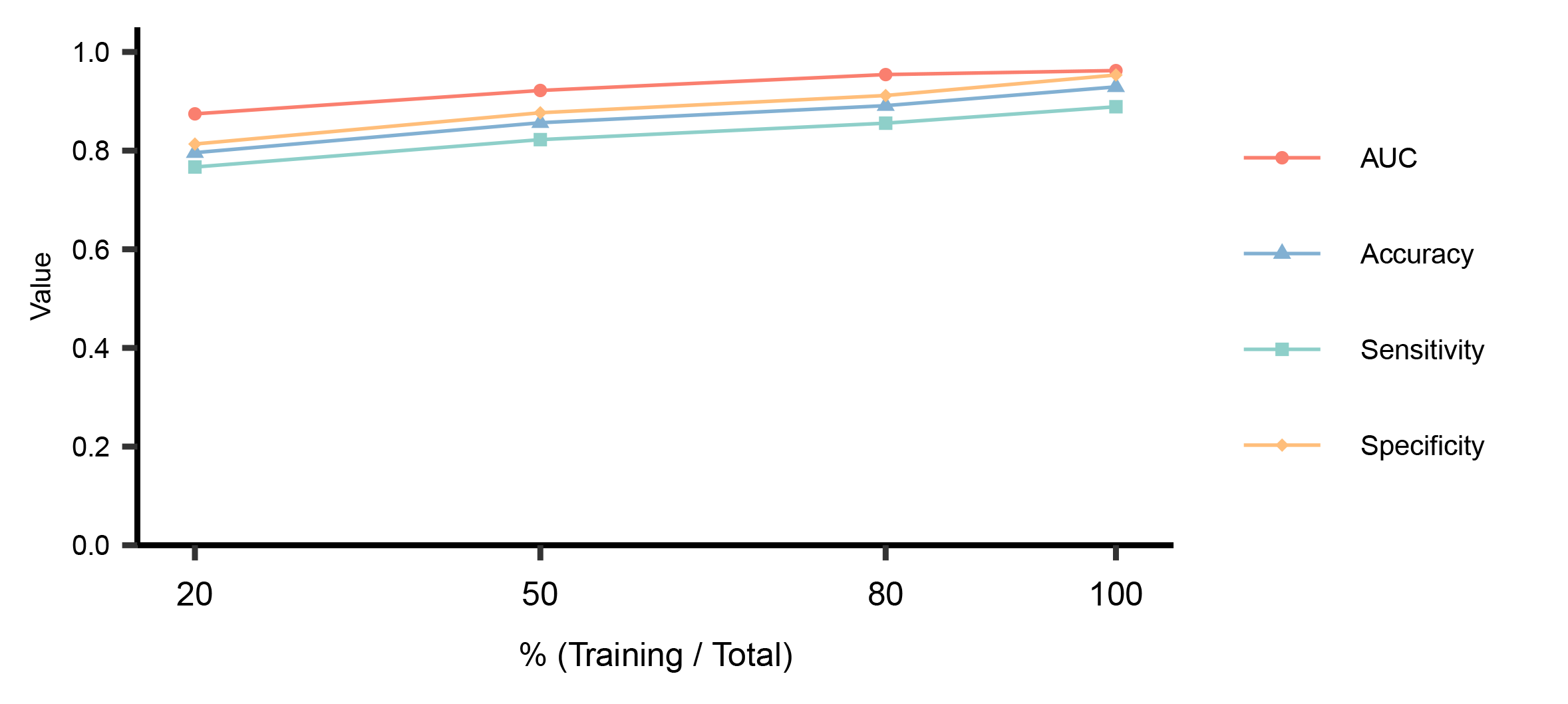


**Figure S4. Performance trends for models with different training set sizes.**

The training set size was gradually reduced from 100% to 80%, 50% and 20% while keeping other settings unchanged.


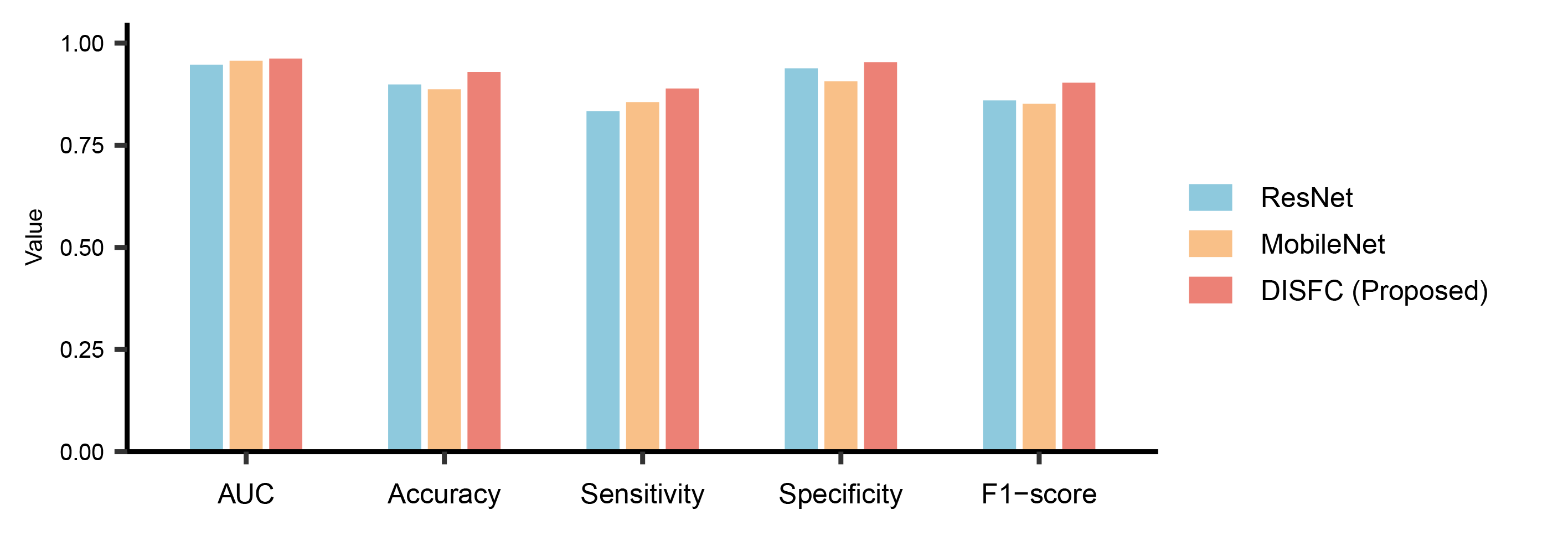


**Figure S5. Performance comparison of models based on different spatial feature extractor backbones.**

The models with ResNet-based, MobileNet-based, and DISFC-based spatial feature extractors were cross-validated under identical settings. Each bar in the chart represents the mean value across folds for respective metrics.


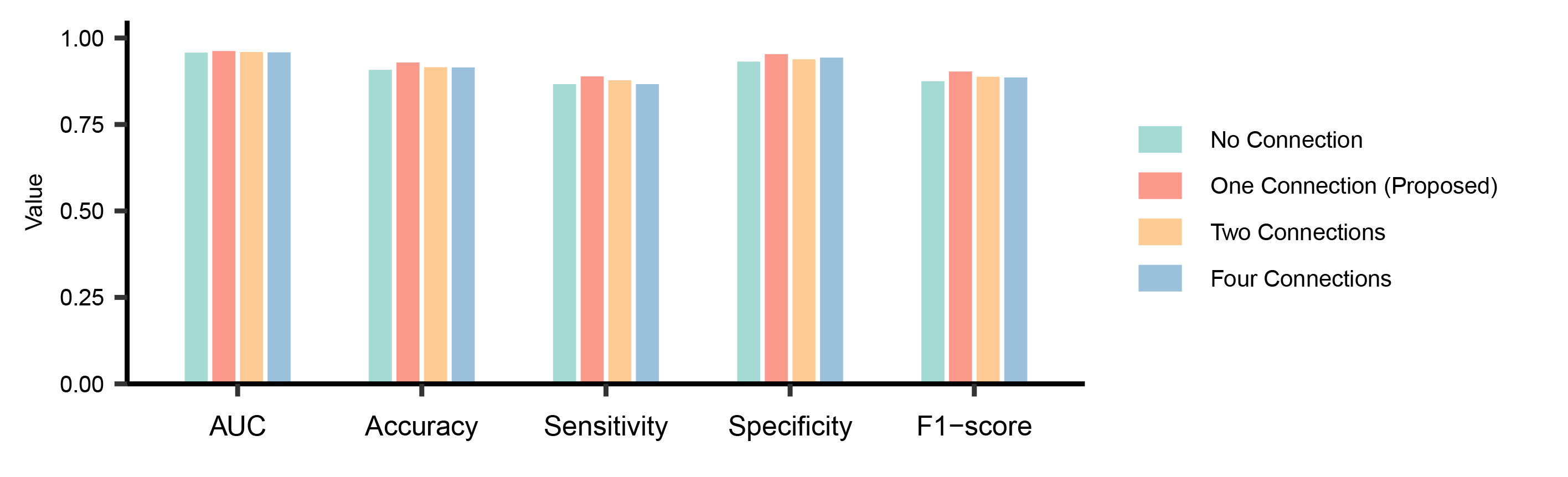


**Figure S6. Performance comparison of models using different residual connection methods.**

The models with 0, 1, 2, and 4 residual connections were cross-validated using the same settings. Each bar in the chart represents the mean value across folds for respective metrics.


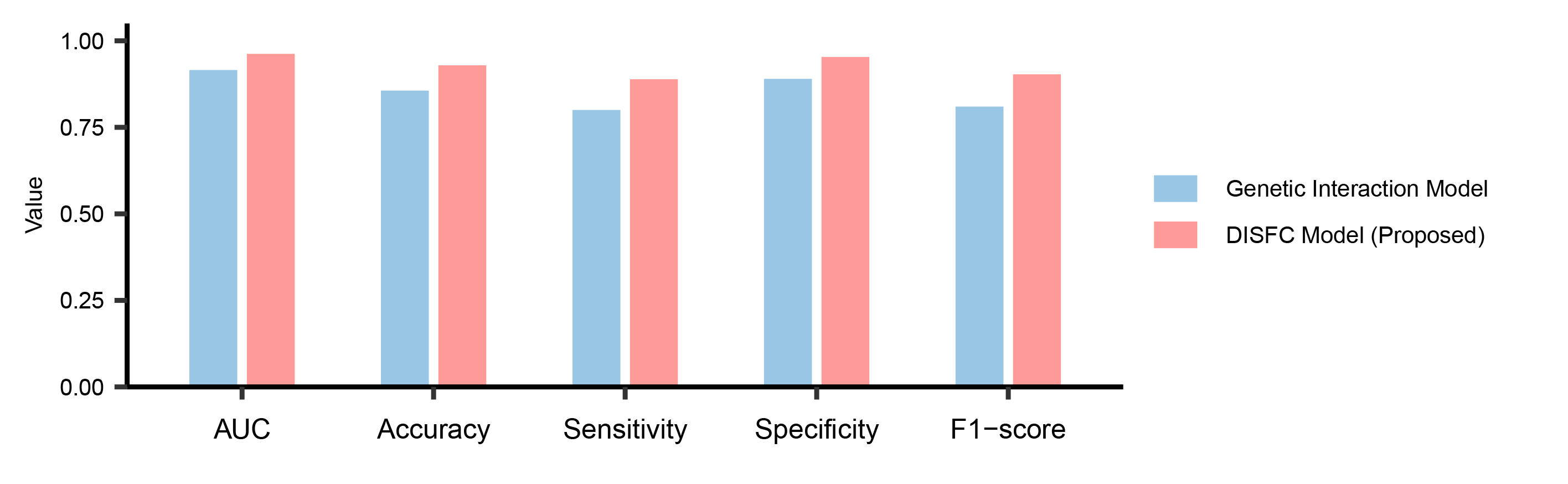


**Figure S7. Performance comparison of the models with and without genetic intra-modal interaction.**

The performance of our DISFC model was compared to the genetic interaction model, which has a similar architecture to DISFC, except for the inclusion of an intra-modal interaction module in the genetic feature extractor. Each bar in the chart represents the mean value across folds for respective metrics.
